# Musikalische Halluzinationen bei einer 92-Jährigen mit Schwerhörigkeit und sozialer Vereinsamung im Rahmen der Corona-Pandemie

**DOI:** 10.1007/s00115-022-01379-y

**Published:** 2022-09-14

**Authors:** Nadia Bieler, Kariem-Noureldin Sharaf, Kristina Adorjan

**Affiliations:** 1 Klinik für Psychiatrie und Psychotherapie, LMU Klinikum, München, Deutschland; 2 Klinik und Poliklinik für Hals-Nasen-Ohrenheilkunde, LMU Klinikum, München, Deutschland

## Hintergrund

Auditorische Halluzinationen sind häufig im Rahmen einer psychotischen Störung anzutreffen. Weitere differenzialdiagnostische Überlegungen sind u. a. dissoziative Störungen, Borderline-Persönlichkeitsstörung, posttraumatische Belastungsstörung oder Störungen organischer Genese, z. B. neurodegenerativ (Parkinson-Demenz, Lewy-Body-Demenz, u. a.), strukturell-neurologisch (Hirnläsionen, Temporallappenepilepsie), oder aus dem HNO-Bereich Presbyakusis.

Das klassische Charles-Bonnet-Syndrom beschreibt das Auftreten von visuellen Halluzinationen bei psychopathologisch unauffälligen Personen ohne zerebrale Läsion mit neu erworbener Visusminderung. Weiteres diagnostisches Kriterium ist der Ausschluss von Delir, demenzieller Entwicklung, Verschlechterung einer affektiven Störung, wahnhaften Störungen, Intoxikationen oder weiteren neurologischen Erkrankungen [6].

Die Deafferenzierungshypothese zur Entstehung auditorischer Halluzinationen postuliert, dass die Reduktion der Reizsignale an den auditorischen Kortex zu einer Erniedrigung der Schwelle für Reizauslösung im Gehirn führt und es folglich zu einem Ungleichgewicht zwischen exzitatorischer und inhibitorischer Neuronenaktivität kommt [2]. Sowohl einfache als auch komplexe auditorische Halluzinationen wurden im Rahmen dieser Störung beschrieben [5]. Die soziale Vereinsamung, in diesem Fall exazerbiert durch die Corona-Pandemie, stellte zusätzlich eine Art „soziale Deafferenzierung“ dar und kann einen weiteren Risikofaktor für die Entwicklung von auditorischen Halluzinationen darstellen [5].

## Kasuistik

Eine rüstige, 92 Jahre alte Patientin stellte sich über die psychiatrische Ambulanz mit seit ca. fünf Monaten anhaltenden, vorwiegend musikalischen Halluzinationen vor. Sie zeigte sich stark belastet und gab an, sich dadurch bedroht zu fühlen.

Die Patientin berichtet, seit einem Sturz rund um Ostern höre sie zunehmend Lieder und Musik, die vor allem nachts aufträten. Sie habe die Polizei rufen müssen, da „die Nachbarn mit der Musik nicht aufhörten“. Es seien Weihnachtslieder, zum Teil auch nationalsozialistische Lieder, die sie seit ihrer Jugend nicht mehr gehört hätte. Außerdem berichtete die Patientin von weiteren Halluzinationen i. S. von Geräuschen (Schleudern einer Waschmaschine) sowie Stimmenhören, teilweise ohne jedoch „zu verstehen, was die Stimmen zueinander sagten“. Mit Hörgeräten sei sie beidseitig seit ca. 6–8 Jahren versorgt worden.

In der MRT fanden sich bis auf mikroangiopathische Läsionen keine Auffälligkeiten. Bei der körperlichen Untersuchung zeigte sich bis auf eine beidseitige Hörstörung ein altersentsprechender Befund. Psychopathologisch war die Patientin wach und allseits scharf orientiert. Aufmerksamkeit, Konzentration und Mnestik waren intakt. Sie zeigte sich formalgedanklich geordnet, keine Hinweise auf eine wahnhafte Störung, keine inhaltlichen Denkstörungen, keine Ich-Störungen. Affektiv zeigte sie sich niedergedrückt, der Antrieb sei erhalten. Psychovegetativ Ein- und Durchschlafstörungen, Appetit regelrecht. Klinisch-neurologisch kein Hinweis auf eine demenzielle Entwicklung (Montreal Cognitive Assessment 27/30 Punkte), kein Hinweis auf eine bradykinetische Gangstörung, die Muskeleigenreflexe waren seitengleich regelrecht auslösbar.

In der HNO-ärztlichen Spiegeluntersuchung zeigte sich eine Verlegung beider Gehörgänge mit Zerumen. Auf Nachfrage gab die Patientin einen Juckreiz im rechten Gehörgang an. Initial gelang die Entfernung nur links und es zeigte sich ein reizloses Trommelfell, rechts entleerte sich am Zeruminalpropf entlang putrides und erheblich fötides Sekret. Wir begannen nach mikrobieller Abstrichdiagnostik eine kalkulierte topische Antibiotikatherapie mit Ciprofloxacin-Ohrentropfen.

**Figure 1:**
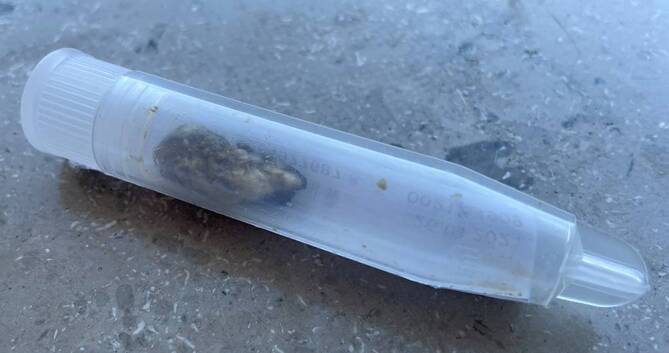

Zwei Tage später gelang rechts die Entfernung eines mehr als 3 cm langen Pfropfens aus Zerumen und Wolle (Abb. 1). Ohrmikroskopisch zeigte sich nun eine entzündete Radikalhöhle. Hierbei handelt es sich um einen bei größeren, chronischen Mittelohrentzündungen durchgeführten Eingriff, bei dem die hintere Gehörgangswand entfernt und zum Mastoid hin eine größere Höhle geschaffen wird.

**Figure 2:**
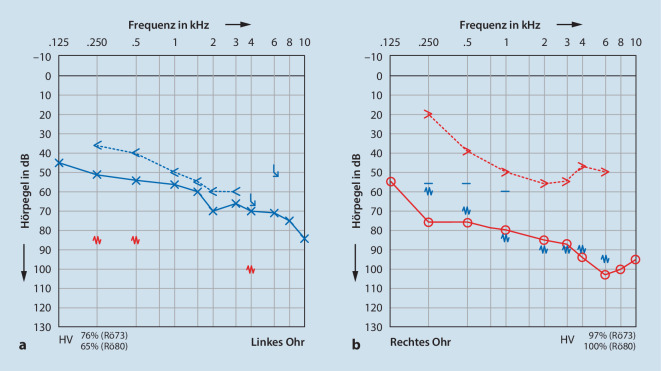

Bei der Kultivierung ergab sich ein Nachweis von Ciprofloxacin-sensiblem P. mirabilis. Im CT Felsenbein zeigte sich ein Z. n. Radikalhöhlenanlage und Tympanoplastik rechts, ohne Nachweis einer Gehörknöchelchenkette oder Rekonstruktion und ohne Hinweis auf knöcherne Arrosion. Linksseitig kamen die Innenohrstrukturen und das Felsenbein unauffällig zur Darstellung. Audiometrisch zeigte sich nach Abheilung der Entzündung eine erhebliche Schwerhörigkeit rechts mehr als links (Abb. 2a, b). Das Knochenleitungsaudiogramm ergab eine annähernd symmetrische Hörkurve. Passend zum ohrmikroskopischen und CT-morphologischen Befund des rechten Mittelohrs zeigte das Audiogramm eine erhebliche kombinierte Schwerhörigkeit rechts mehr als links. Das korrespondierende Sprachaudiogramm zeigte rechts ein Einsilberverstehen von 60 % bei 110 dB im Freiburger Sprachtest, links 65 % bei 95 dB und 90 % bei 110 dB.

## Diagnose

Die neu aufgetretenen auditorischen Halluzinationen bei einer zuvor psychisch gesunden 92-jährigen Patientin deuteten auf eine organische Genese hin. Nach Ausschluss weiterer Differenzialdiagnosen (wahnhafter Störung, demenzieller Entwicklung, struktureller Hirnläsionen u. a.) und Sichtung aller Befunde stellten wir die Diagnose eines auditorischen Charles-Bonnet-Syndroms.

Eine ursächliche Therapie erfolgte mit Entfernung des Fremdkörpers und antibiotischer Behandlung im rechten Gehörgang, zur Symptomlinderung setzten wir ein niederpotentes Neuroleptikum (Pipamperon mit 40 mg zur Nacht) an. Bei Entlassung ca. 6 Wochen später gab die Patientin eine deutliche Reduktion der Intensität der auditorischen Halluzinationen an. Die Musik sei „weiter weg und leiser geworden“, der Schlaf habe sich deutlich gebessert. Ca. 6 Monate nach Entlassung zeigte sich die Patientin auf Nachfrage beschwerdefrei.

## Diskussion

Musikalische Halluzinationen stellen ein häufiges Phänomen bei Menschen höheren Alters dar. Risikofaktoren für ein auditorisches Charles-Bonnet-Syndrom sind ein weibliches Geschlecht, Schwerhörigkeit, Alter über 60 Jahre und soziale Isolation [3].

Die Inzidenz auditorischer Halluzinationen bei Schwerhörigkeit ist relativ hoch, bei einer Studie mit 829 Probanden betrug sie 16 % und korrelierte mit dem Grad der Schwerhörigkeit [4]. Verschiedene Behandlungsoptionen stehen zur Verfügung, wenn möglich soll jedoch eine kausale Behandlung erfolgen. In der Gruppe der Hyp- bzw. Presbyakusis-Patienten können eine Aufklärung zur Ätiologie der Krankheit, eine Optimierung der Hörgerätversorgung sowie der Einsatz externer auditorischer Stimuli (i. S. eines Maskings ähnlich der Behandlung eines Tinnitus) eine Erleichterung bringen [1].

## Fazit für die Praxis

Eine gründliche organische Abklärung insbesondere des Hörorgans ist bei neu aufgetretenen auditorischen Halluzinationen zwingend zu empfehlen.
